# The Yin and Yang of Type 1 Regulatory T Cells: From Discovery to Clinical Application

**DOI:** 10.3389/fimmu.2021.693105

**Published:** 2021-06-10

**Authors:** Ece Canan Sayitoglu, Robert Arthur Freeborn, Maria Grazia Roncarolo

**Affiliations:** ^1^ Division of Hematology, Oncology, Stem Cell Transplantation and Regenerative Medicine, Department of Pediatrics, Stanford School of Medicine, Stanford, CA, United States; ^2^ Institute for Stem Cell Biology and Regenerative Medicine (ISCBRM), Stanford School of Medicine, Stanford, CA, United States; ^3^ Center for Definitive and Curative Medicine (CDCM), Stanford School of Medicine, Stanford, CA, United States

**Keywords:** stem cell, transplantation, immunotherapy, immune tolerance, Tr1 cells

## Abstract

Regulatory T cells are essential players of peripheral tolerance and suppression of inflammatory immune responses. Type 1 regulatory T (Tr1) cells are FoxP3^-^ regulatory T cells induced in the periphery under tolerogenic conditions. Tr1 cells are identified as LAG3^+^CD49b^+^ mature CD4^+^ T cells that promote peripheral tolerance through secretion of IL-10 and TGF-β in addition to exerting perforin- and granzyme B-mediated cytotoxicity against myeloid cells. After the initial challenges of isolation were overcome by surface marker identification, *ex vivo* expansion of antigen-specific Tr1 cells in the presence of tolerogenic dendritic cells (DCs) and IL-10 paved the way for their use in clinical trials. With one Tr1-enriched cell therapy product already in a Phase I clinical trial in the context of allogeneic hematopoietic stem cell transplantation (allo-HSCT), Tr1 cell therapy demonstrates promising results so far in terms of efficacy and safety. In the current review, we identify developments in phenotypic and molecular characterization of Tr1 cells and discuss the potential of engineered Tr1-like cells for clinical applications of Tr1 cell therapies. More than 3 decades after their initial discovery, Tr1 cell therapy is now being used to prevent graft versus host disease (GvHD) in allo-HSCT and will be an alternative to immunosuppression to promote graft tolerance in solid organ transplantation in the near future.

## Introduction

More than 30 years ago, studies in a severe combined immunodeficiency (SCID) patient that had undergone fully mismatched allogenic hematopoietic stem cell transplantation (allo-HSCT) led to the groundbreaking discovery of type 1 regulatory T (Tr1) cells (1). In this patient, mixed chimerism with donor T cells and host antigen presenting cells (APCs) and B cells was established in the absence of immunosuppression. Donor-derived T cell clones had the ability to respond to host allo-antigens *in vitro*, but no graft-versus-host disease (GvHD) was observed *in vivo*, suggesting an active mechanism of tolerance. In subsequent studies, it was discovered that the host-reactive T cell clones isolated from the transplanted patient produced IL-2, IFN-γ and GM-CSF, but not IL-4, suggesting a role for these cytokines in the observed peripheral tolerance (2). Shortly after the cloning of human IL-10 (3), a second transplanted SCID patient was found to have CD4^+^ T cells specific for host allo-antigens that secreted high levels of IL-10 (4). Further investigation in healthy donors and mice led to the finding of tolerance-inducing, high IL-10-secreting cells that were named Tr1 cells (5).

Tr1 cells can be defined as CD4^+^Foxp3^-^ regulatory T cells that promote tolerance and dampen inflammatory immune responses. At resting state, Tr1 cells can be found in the spleen or periphery [reviewed in (6)]. Upon antigen-specific TCR activation, Tr1 cells secrete high levels of IL-10 and TGF-β that suppress proliferation of effector T cells and pro-inflammatory cytokine production by APCs. In addition to IL-10 and TGF-β secretion, Tr1 cells also suppress effector T cell responses *via* engagement of CTLA-4/CD80 or PD-1/PD-L1 (7). Additionally, activated Tr1 cells secrete granzyme B and exert cytotoxicity against myeloid APCs (8), thereby preventing naïve T cell priming (9). Thanks to their ability to prevent production of inflammatory cytokines by APCs and suppress effector T cells, Tr1 cells act as a bridge between innate and adaptive immune responses.

## Generation of Human Tr1 Cells

*Ex vivo* isolation of Tr1 cells from peripheral blood CD4^+^ T cells of healthy donors results in poor yields since Tr1 cells in circulation are in low quantities [1-10% of peripheral blood memory CD4^+^ T cells (10)]. Tr1-rich cell products and antigen-specific Tr1 cell clones have been expanded *in vitro* using a feeder cell mixture or other artificial APCs. With the discovery and use of tolerogenic dendritic cells producing high levels of IL-10 (DC-10) (11) as stimuli for CD4^+^ T cells, an *in vitro* method to generate alloantigen-specific Tr1 cells was successfully developed for clinical applications. Another product comprised of engineered polyclonal Tr1-like cells has been developed and tested in pre-clinical models showing promising results to prevent GvHD and exert anti-leukemia responses (12–14). All methods are reviewed in detail elsewhere (6) but in summary, these protocols can be grouped as follows:

### Peripheral Blood-Derived Tr1 Cell Lines and Clones

In early studies bulk T cell populations enriched for Tr1 cells were generated by culturing peripheral blood mononuclear cells (PBMC) or CD4^+^ T cells with allogeneic monocytes and exogenous IL-10 (5). After the initial polyclonal expansion, T cell clones were cultured as single cells and expanded in the presence of irradiated feeder cells containing third party PBMCs and the allogeneic EBV-immortalized cell line JY to obtain antigen-specific Tr1 cells. Polyclonal Tr1 cell lines could also be generated and expanded using mouse L cells expressing hCD32, hCD58 and hCD80 in the presence of anti-CD3 antibody, IL-10 and IFN-a (15). In another study, human ovalbumin-specific Tr1 cell clones were generated by using Drosophila-derived APCs (16). Overall, peripheral blood Tr1 cells could be isolated and expanded *in vitro* in the presence of feeder cells.

### *In Vitro* Tr1 Generation by Tolerogenic DC Stimulation

Immature monocyte-derived dendritic cells (iDCs) enabled the generation of Tr1 cells from naïve CD4^+^ T cells *in vitro* and this differentiation was IL-10-dependent (17). Following these findings, the discovery of DC-10 – an IL-10-secreting, ILT4^+^ HLA-G^+^ DC population generated *in vitro* from monocytes in the presence of IL-10 – allowed the optimization of an *in vitro* allo-antigen-specific Tr1 generation protocol (11). Recently, both *ex vivo*-generated and *in vivo* DC-10 were shown to express CD141 and CD163, enabling *ex vivo* isolation of DC-10 population from peripheral blood (18). Naïve CD4^+^ T cells isolated from healthy donor PBMCs can be cultured with allogeneic DC-10 to generate anergic, alloantigen-specific Tr1-enriched cell populations *in vitro* and can be safely used for cell therapy (19). Another Tr1-enriched cell therapy product, T10, was generated by co-culturing T cells of transplant recipients with organ donor DCs to promote tolerogenic Tr1 generation (20). This preclinical study confirmed the potential benefit of T10 to prevent graft rejection, as explained in later sections. A similar strategy is now being used to generate the T-allo10 cell therapy product to prevent GvHD in allo-HSCT. Briefly, peripheral blood monocyte-derived DC-10 cells of a patient undergoing allo-HSCT and CD4^+^ T cells of the stem cell donor are isolated and co-cultured *ex vivo* in the presence of exogenous IL-10 to generate patient alloantigen-specific tolerogenic T-allo10 cells. This Tr1-rich cell therapy product T-allo10 is currently in phase I clinical trial (Clinicaltrials.gov identifier NCT03198234), explained in later sections.

### Next Generation of Engineered Tr1-Like Cells: CD4^IL-10^

CD4^IL-10^ are engineered Tr1-like cells and are generated by transducing human peripheral blood CD4^+^ T cells with a lentiviral vector over-expressing human *IL10 (hIL10)*. CD4^IL-10^ display a Tr1 cytokine production profile (IL-10^+^IFN-γ^+^IL-2^-^IL-4^-^), phenotypic markers (CD2^+^CD18^+^CD226^+^), suppressive properties, and cytotoxicity against myeloid cells. CD4^IL-10^ also prevent xeno-GvHD in humanized mouse models (12, 13) and kill primary acute myeloid leukemia blasts (13, 14). Interestingly, CD4^IL-10^ upregulate cytotoxicity-related genes correlated with constitutive expression of IL-10 (our unpublished data). In contrast to other methods which produce low yields of poorly proliferating Tr1 cell products, polyclonal CD4^IL-10^ grow and expand *in vitro* for several weeks, providing larger quantities of Tr1-like cells. Accordingly, CD4^IL-10^ are promising candidates to be used in clinic in near future for prevention of GvHD and promotion of anti-leukemia responses in acute myeloid leukemia patients going through allo-HSCT.

## Biological Characterization of Human Tr1 Cells

### Phenotype

Tr1 cells were hard to identify for many years due to the lack of specific surface markers. The discovery of LAG3 and CD49b expressed on both murine and human memory CD4^+^ Tr1 cells enabled the isolation and characterization of Tr1 populations from peripheral blood of healthy donors (10). Since then, many other studies confirmed the expression of these proteins on murine (21–29), non-human primate (30) and human (18, 31–44) Tr1 cell populations.

It is important to note that CD49b and LAG3 are not exclusively expressed on Tr1 cells and are not sufficient to identify Tr1 cells alone. This is mainly due to the fact that LAG3 is also expressed by activated T cells and CD49b is expressed on memory T cell populations (45). Nevertheless, our data support that when LAG3^+^CD49b^+^ memory T cells are purified, the regulatory function expected of Tr1 cells is confined to this population. Single-cell RNA sequencing analysis of IL-10-producing CD4^+^FoxP3^-^ cells revealed that Tr1 cells can be differentiated from other IL-10-producing CD4^+^ T cells by their expression of co-inhibitory receptors (CIRs) in addition to LAG3 and CD49b (46). High IL-10 secretion was correlated with high granzyme B as well as expression of CIRs LAG3, TIGIT, TIM3 and PD-1 in CD4^+^FoxP3^-^ T cells. Only the CIR-rich IL-10-secreting splenic T cells had suppressive capacity as shown by *in vivo* models of colitis. Thus, IL-10 alone is not a factor in determining Tr1 identity but the combination of IL-10 with granzyme B and CIRs along with suppressive function frame the Tr1 population definition. In conclusion, on top of LAG3 and CD49b, Tr1 phenotyping should be supported by other surface markers such as PD-1, CTLA-4, ICOS, TIM-3, TIGIT, CD226, CD73 and CD39 [reviewed in (47)].

### Molecular Characterization

The search for key transcription factors playing a role in Tr1 cell generation and function has been hampered until recently by the difficulty of purifying Tr1 cells and distinguishing them from other IL-10-producing T cells. Unlike Foxp3^+^ regulatory T cells (Tregs), Tr1 cells currently do not have a defined master regulator that can be used as a lineage-determining transcription factor. The difference between Tregs and Tr1 cells was unequivocally demonstrated when CD4^+^ T cells from IPEX patients carrying loss-of-function *FOXP3* mutations could differentiate *in vitro* into functional Tr1 cells (48).

Transcription factors that play a role in anergy induction and IL-10 production were among the first candidates to be investigated for their relevance in Tr1 biology and function. For instance, EGR-2, a key transcription factor for T cell anergy, was found to be expressed in Tr1 cells (49, 50), and EGR2 overexpression in naïve CD4^+^ T cells caused elevated LAG3 expression and IL-10 production (10, 51). *In vitro* culture of murine T cells with IL-27 and TGF-β drove the differentiation of IL-10 producing T cells (52). Notably, there are several transcription factors that induce IL-10 production upon IL-27 treatment in murine CD4^+^ T cells. In a recent study covering transcriptional and epigenetic profiling of murine Th cell subsets, Blimp-1 (encoded by the *Prdm1* gene) and Maf were demonstrated to be two transcription factors driving the expression of genes induced with IL-27, including IL-10 in Tr1 cells (53). *Prdm1* and *Maf* double knock-out led to spontaneous colitis, mimicking the phenotype observed in IL-10 knockout mice (53). IL-27 signals through EGR-2 to induce IL-10 production by Blimp-1 in CD4^+^ T cells (54), and EGR-2 is necessary for IL-27-induced Blimp-1-dependent IL-10 induction (51). Blimp-1 is required for high IL-10 secretion, not only by Tr1 cells but also by other CD4^+^ T cells (55, 56) and CD8^+^ cytotoxic T lymphocytes (57). Blimp-1 deficiency results in markedly less IL-10 production by CD4^+^ T cells, while its overexpression promotes Tr1 generation (58). Recently, eomesodermin (Eomes) was demonstrated to coordinate with Blimp-1 to drive Tr1 polarization, and Eomes was critical for protection against GvHD (59). Importantly, T-bet was also crucial for Eomes-mediated Tr1 differentiation. As previously mentioned, c-Maf also contributes to IL-27-induced IL-10 production by Tr1 cells. The aryl hydrocarbon receptor (AHR) also contributes to Tr1 differentiation (60). Interestingly, this has been shown to occur through the formation of AHR/c-Maf heterodimers (61) and through metabolic regulation (25). A recent study also revealed that a cytokine, activin-A, induced an IRF4/AHR transcriptional network in human cells to drive Tr1 differentiation, and adoptive transfer of activin-A-induced Tr1 cells protected against asthma in humanized mouse models (62). The role for IRF4 was further shown in mouse models of infection in which ITK signaling through Ras/IRF4 promoted Tr1 differentiation *in vivo* (24). Additionally, transcription factors IRF1 and BATF were stated as ‘pioneering factors’ that have substantial roles in murine Tr1 differentiation and function (63). Both IRF1 and BATF are induced upon IL-27 stimulation and are required for chromatin accessibility of Tr1-associated genes as shown by ATAC-seq analysis (63). Taken together, downstream signaling of IL-27 shapes the epigenetic and transcriptomic requirements for Tr1 generation and function in murine T cells.

Continuous expression of *IL10* in human peripheral blood CD4^+^ T cells is essential to drive Tr1 cell differentiation; however, IL-10 alone is not a lineage-determining factor since it can be secreted by many cells of the immune system. IL-10 producing CD4^+^ T cells are a heterogenous population, but Tr1 cells can be characterized with the expression of CIRs (46). In this study, IL-10-secreting CD4^+^FoxP3^-^ T cells isolated from the spleens and small intestines of mice had tissue-dependent transcriptional profiles. Single-cell RNA sequencing showed that IL-10-producers from the spleen were more diverse in terms of their transcriptional profile compared to the intestine. Id2 and Bhlhe40 are additional transcription factors found highly expressed in IL-10-producing CD4^+^ CIR-rich cell subsets but their specific roles in these cell subsets are yet to be determined (46). In a different study, Bhlhe40 was required to repress IL-10 expression in *Mycobacterium tuberculosis* infected mice (64). Interestingly, *Yu* et al. showed that Bhlhe40 behaved as a switch between anti and proinflammatory states of Th1 cells, regulating IL-10 expression depending on different immunological cues (65). Thus, the role of Bhlhe40 in Tr1 generation needs further investigation.

While the aforementioned transcription factors revolve around IL-10 regulation, the IL-10 Receptor (IL-10R) is also essential in Tr1 induction *in vivo* as well as Tr1 generation *in vitro* (66). IL-10R signals through STAT3 and p38/MAPK, resulting in activation of multiple immune-related pathways. STAT3 is also hyperphosphorylated in activated peripheral blood Tr1 cells (67). The IL-10/STAT3 axis might be essential in regulation of transcription factors required for Tr1 generation and function, contributing to the maintenance of peripheral tolerance (68).

TCR stimulation is essential for Tr1 suppressive function both *in vitro* and *in vivo* (9). ITK, a kinase activated downstream of TCR activation modulates Tr1 generation in murine and human CD4^+^ T cells. In ITK knock-out mice, IL-10 production is impaired and the suppressive function of ITK-deficient cells can be reversed by IRF4 expression. As mentioned above, ITK is responsible for the Ras/IRF4 activation that contributes to CD4^+^ T cell development into CD49b^+^LAG3^+^ Tr1 cells (24). As with canonical TCR stimulation for T cell activation, costimulatory and coinhibitory signals have been demonstrated to affect Tr1 cell differentiation. In one case, stimulation of murine naïve CD4^+^ T cells with anti-CD3 in the presence of soluble PD-L1 produced a substantial proportion of Tr1-like cells (69). Similarly, activation of human CD4^+^ T cells with anti-CD3 in the presence of anti-CD46 led to downregulation of IL-2 and gave rise to Tr1 cells (70, 71). Interestingly, this effect is not seen in T cells from MS patients (72). Costimulation of human naïve CD4^+^ T cells through CD55 also produces highly suppressive Tr1 cells (73).

Overall, these data suggest that several distinct transcription factors and signaling pathways play roles in differentiation, generation and activation of Tr1 cells through IL-27 stimulation, regulation of IL-10 gene expression or through altering suppressive and cytotoxic functions of Tr1 cells. To date, no single transcription factor has been proven to be sufficient to lead to Tr1 generation alone and their dynamic interactions within mouse or human Tr1 cells require further investigations.

### Cytotoxic Properties of Tr1 Cells

In addition to their well-known suppressive function, both peripheral and engineered Tr1 cells (CD4^IL-10^) are able to recognize and kill cells of myeloid origin (8, 12–14). Constitutive expression of *hIL10* in human CD4^+^ T cells results in upregulation of cytotoxicity-related genes (our unpublished data). Tr1 cells and CD4^IL-10^ express high amounts of granzyme B along with CD2, CD18 and CD226 that contribute to myeloid cell killing (8, 12, 13). CD2 binds to CD54, CD18 binds to CD58, and CD226 binds to CD112 and CD155 found on target cells, enhancing adhesion and sending activating signals. Myeloid target cell killing is dependent on granzyme B expression by CD4^IL-10^ and HLA class I expression on target cells (8, 13). In addition to myeloid cell lines and primary adult acute myeloid leukemia, we recently showed that CD4^IL-10^ can also kill pediatric AML blasts (14). We observed that pediatric AML differ in their sensitivity to CD4^IL-10^ mediated killing and group as sensitive, intermediate-resistant, or resistant. RNA sequencing of different groups of pediatric AML showed that sensitive and resistant blasts have distinct transcriptional profiles. *CD200*, a prognostic factor for AML (74), was differentially expressed on the resistant pediatric AML. The receptor for CD200, CD200R1, is expressed on T cells and functions as an inhibitory receptor contributing to diminished cytotoxic responses (14). Thus, it is possible that resistant pediatric AML have signature molecules upregulated on their surface that can be used as predictive markers to select patients with AML suitable for CD4^IL-10^ cell therapy. Tr1 cells or engineered Tr1-like cells can enhance anti-tumor response once delivered to a leukemia patient undergoing allo-HSCT, targeting residual tumor cells that would otherwise cause relapse. While exerting anti-tumor activity, Tr1 cells also promote tolerance and thus provide a combinatorial benefit to the patient.

## Clinical Application of Tr1 Cells

### Suppressive Roles in Transplantation

The ability of Tr1 cells to maintain tolerance in organ transplantation has been demonstrated in many preclinical studies. *Battaglia* et al. showed that rapamycin plus IL-10 treatment induced Tr1 cells that modulated long term tolerance *in vivo* and prevented allograft rejection following islet transplantation (75). Notably, antigen-specific Tr1 cells were found to be more potent than polyclonal Tr1 (76). Interestingly, Tr1 cells localized in the spleen maintained long-term tolerance (77). A potential advantage of Tr1 cell therapy compared to traditional immune suppression was recently demonstrated: Tr1 cell therapy did not alter the anti-viral response in islet transplant models in mice, nor did viral infections change Tr1 efficacy in maintaining transplant tolerance (78). Recently, graft-infiltrating M2 macrophages were shown to induce Tr1-mediated tolerance after islet cell transplantation in mouse models (79). Accordingly, CD206^high^ IL-10-producing macrophages infiltrated the graft of G-CSF/rapamycin-treated tolerant mice and *in vivo* depletion of phagocytic cells abrogated graft tolerance and Tr1 induction (79). Taken together, these preclinical studies show the crosstalk between Tr1 cells and other immune cells and demonstrate that Tr1 cells promote graft tolerance in islet transplantation while preserving anti-viral immunity.

**Figure 1:**
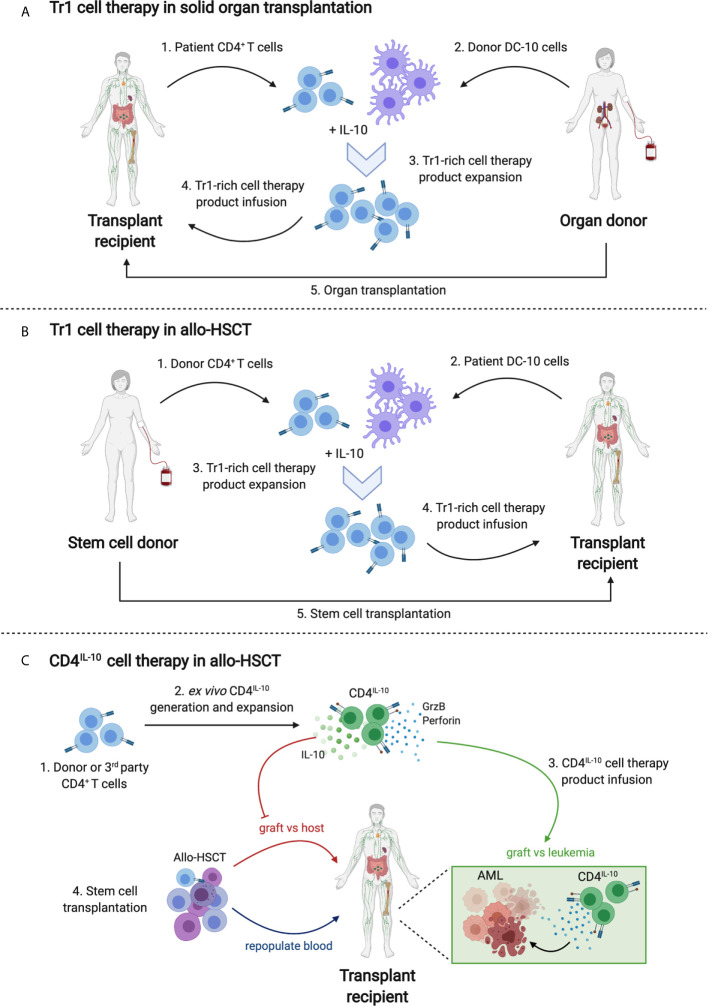
Tr1 cell therapy strategies in transplantation. **(A)** Tr1 cell therapy in solid organ transplantation. Patient CD4^+^ T cells (1) and donor tolerogenic DC-10 cells (2) will be isolated prior to organ transplantation. A Tr1-rich cell therapy product will be expanded by culturing CD4^+^ T cells and DC-10 in the presence of exogenous IL-10 (3). The alloantigen-specific Tr1-rich cell therapy product will be phenotyped and tested *ex vivo* for suppressive capacity and will be infused into the transplant recipient (4). Concurrently or a day after infusion, organ transplantation will take place (5). **(B)** Tr1 cell therapy in allo-HSCT. Donor CD4^+^ T cells (1) and patient-derived tolerogenic DC-10 cells (2) will be isolated prior to HSCT. Steps (3) and (4) will be followed as in part **(A)**. A day after infusion, HSCT will take place (5). **(C)** CD4^IL-10^ cell therapy in allo-HSCT. In this example, the transplant recipient is an acute myeloid leukemia patient. Stem cell donor or 3^rd^ party healthy donor CD4^+^ T cells will be isolated (1) and transduced with *hIL10* lentivirus to generate CD4^IL-10^ cells (2). After *ex vivo* expansion and quality control, CD4^IL-10^ cells will be infused to the transplant recipient (3) concurrently with allo-HSCT (4). While donor stem cells repopulate blood, donor T cells promote graft versus host disease (GvHD). CD4^IL-10^ cells prevent GvHD by secretion of IL-10 and promote graft versus leukemia (GvL) response *via* granzyme B/perforin mediated cytotoxicity against residual AML cells.

In solid organ transplantation, host immune cells attack donor organs, leading to transplant rejection. To prevent this immune response, transplant recipients are obliged to take life-long immunosuppressive treatments that have severe negative impacts, including recurrent severe infections. A key approach to replace or minimize immunosuppressive treatments is to induce donor-specific tolerance. A clinical product called T10 was developed to optimize the production of Tr1-enriched cell products in patients undergoing kidney transplants, aiming to prevent graft rejection (20, 80) (**Figure 1A**). The T10 product is generated by culturing donor DC-10 with patient CD4^+^ T cells in the presence of exogenous IL-10. CD4^+^ T cells isolated from patients undergoing dialysis were able to reach sufficient numbers of *ex vivo* expanded T10 cells that maintained suppressive abilities stably after cryopreservation. In parallel, phenotyping was done periodically with peripheral blood Tr1 cells of patients going through immunosuppression to understand potential effects of immunosuppressive treatments on Tr1 cells, since planned T10 infusion would take place in patients receiving immunosuppressive treatments. The phenotype of Tr1s from transplant patients were comparable to healthy controls and optimal times for T10 infusion were considered to be at time zero and around 36 weeks after transplantation. These pre-clinical studies were essential to optimize Tr1 medicinal product quality and demonstrate the potential for transplant patients, but no product infusion has been done yet. Despite the promising results in pre-clinical studies, the role of Tr1 in solid organ transplantation is a field anticipating in-patient trials.

### Tr1 Therapy in the Context of allo-HSCT

Many leukemia patients are dependent on successful allo-HSCT for treatment of their hematological malignancies and reconstitution of a healthy immune system. Allo-HSCT is also the only cure for patients with inherited blood disorders, such as SCID and beta thalassemia. Following the initial studies that demonstrated Tr1-mediated tolerance after mismatched allo-HSCT in SCID patients (1, 2, 4), Tr1 cells were also shown to play a role in thalassemic patients with persistent mixed chimerism after allo-HSCT (81). In a proof-of-concept clinical study, the ALT-TEN trial, twelve patients with hematological malignancies undergoing haplo-HSCT were given IL-10-anergized donor T cells (IL10-DLI) containing Tr1 cells to support immune reconstitution in the absence of severe GvHD (32). 4 patients had positive outcomes, long term disease remission and gene expression profiles of these patients’ cells were associated with tolerance (32). This study led the way to the use of Tr1 cell therapy in allo-HSCT recipients.

In the current T-allo10 clinical trial (Clinicaltrials.gov identifier NCT03198234), the IL-10-anergized donor T cells specific for the host allo-antigens are generated using an optimized protocol. Donor CD4^+^ T cells are co-cultured with host-derived DC-10 to ‘educate’ the T cells to become anergic to the host allo-antigens and to generate alloantigen-specific Tr1 cells (**Figure 1B**). The T-allo10 cell product contains up to 15% Tr1 cells. T-allo10 cells are infused one day before the transplant to patients undergoing related or unrelated mismatched allo-HSCT. The goal of this study is to show the safety, unravel the maximum tolerated dose and demonstrate the capacity of the T-allo10 cells to prevent GvHD and promote long term tolerance in patients receiving mismatched HSCT. Thus far, the preliminary analysis shows that the therapy is well tolerated, with long-term persistence of Tr1 cells (*Chen* et al., unpublished data). Recently, the T-allo10 trial has also been expanded to patients undergoing alpha-beta T cell-depleted haplo-HSCT to boost immune cell reconstitution and help prevent GvHD (Clinicaltrials.gov identifier NCT04640987). Overall, the T-allo10 clinical trial will be a first step in determining *in vivo* safety and efficacy of alloantigen-specific tolerogenic Tr1 cells that can be further used in other clinical settings beyond allo-HSCT.

Beyond these studies, there are also preclinical studies investigating the method of *in utero* HCT for prevention of congenital hematological diseases, but the injection window and technical difficulties have not been overcome yet. Nevertheless, it was recently demonstrated that treatment with tolerance-inducing Tregs and Tr1 cells prevented GvHD and promoted long-term multilineage chimerism in mice receiving *in utero* HCT (82).

### Other Immune Mediated Diseases

Tr1 cells play important roles in suppressing autoimmunity and it has been reported that Tr1 function may be impaired in diseases like type I diabetes, colitis, and multiple sclerosis (MS) (83). In patients with refractory Crohn’s disease, ovalbumin-specific Treg (OVA-Tregs) cells isolated from patients’ blood and restimulated with ovalbumin have been reinfused intravenously (84). This study (Crohn’s And Treg Cells Study- CATS1) demonstrated that Treg infusions were well-tolerated and reduced pathology in some patients. Tr1 cells can modulate inflammasome activity in macrophages *via* secretion of IL-10 (28), which can be a potential approach in treating inflammatory bowel disease (IBD). It was also recently revealed that the phenotype of Tr1 cells isolated from the gut of patients with Crohn’s disease or ulcerative colitis was not different from that of healthy individuals after *ex vivo* expansion. In addition, these Tr1 cells were able to secrete IL-22, promoting barrier function in intestinal epithelial cells (85). Collectively, Tr1 cell therapy is a promising approach for treating IBD.

### Considerations for Future Clinical Applications

Treg cell therapies for autoimmune disorders and transplant patients is an area of increasing interest. Despite the abundance of promising preclinical studies, several hurdles remain for the clinical application of Treg cell therapies [reviewed in (86, 87)]. In the current clinical trial in allo-HSCT, the manufactured alloantigen-specific T-allo10 cell product is only enriched in Tr1 cells. The lack of purity represents a significant limitation to the use of this cell product in other clinical settings. Nevertheless, administration of tolerogenic Tr1-enriched cell products could increase graft tolerance in solid organ transplant patients, comparable to their tolerogenic effect in allo-HSCT. Antigen-specific Tr1 cells can also be generated for known self-antigens causing autoimmune disorders, like early-onset type 1 diabetes, to prevent autoreactive T cells from destructing islet cells (88).

CD4^IL-10^, engineered Tr1 cells that overexpress *hIL10*, is a pure cell therapy product which should be considered for future clinical applications. Preclinical studies demonstrate that CD4^IL-10^ not only share the functional properties of Tr1 cells but also can grow and expand in abundance. The bidirectional lentiviral vector used for concurrent expression of IL-10/delta-NGFR has been recently updated to meet FDA requirements (*Liu* et al, unpublished data). Polyclonal CD4^IL-10^ cells can provide a GMP-compliant cell therapy option for leukemic patients undergoing allo-HSCT in the near future (**Figure 1C**). The future generation of antigen-specific engineered Tr1-like cells will also broaden their clinical applications to other immune mediated diseases.

## Concluding Remarks

In the 33 years since their discovery, Tr1 cells have been shown to be essential in peripheral tolerance in transplant settings, chronic infections, and autoimmune disorders. While it was challenging to isolate, expand, and identify these cells, we now have more information than ever regarding their phenotype, function, and *ex vivo* expansion. Multiple single cell level -omics analyses such as single cell RNA sequencing and ATAC sequencing are advancing our understanding of key molecular mechanisms regulating Tr1 differentiation and function. Alloantigen-specific Tr1-enriched cell products in clinical trials will yield insights on safety and efficacy of these cells and pave the way for their use in other clinical settings such as solid organ transplantation and tissue stem cell therapies. Furthermore, refined methods to generate and expand polyclonal and antigen-specific Tr1 cells will allow their application in autoimmune and chronic inflammatory disorders in the near future.

## Author Contributions

All authors contributed to the article and approved the submitted version.

## Conflict of Interest

The authors declare that the research was conducted in the absence of any commercial or financial relationships that could be construed as a potential conflict of interest.
